# Protocol for a randomized controlled trial evaluating the effect of *Hibiscus syriacus* L. flower extract on sleep quality

**DOI:** 10.3389/fnut.2023.1169193

**Published:** 2023-04-20

**Authors:** Yujin Choi, Yu Hwa Park, Changsop Yang, Do Hoon Kim, Kye Wan Lee, Mi Young Lee

**Affiliations:** ^1^KM Science Research Division, Korea Institute of Oriental Medicine, Daejeon, Republic of Korea; ^2^R&D Center, Dongkook Pharm. Co., Ltd., Suwon, Republic of Korea; ^3^KM Convergence Research Division, Korea Institute of Oriental Medicine, Daejeon, Republic of Korea

**Keywords:** *Hibiscus syriacus* L. flower, sleep quality, functional food, dietary supplement, clinical protocol, randomized controlled trial

## Abstract

**Introduction:**

*Hibiscus syriacus* L. flower (HSF) is a food ingredient commonly used for tea, and previous animal studies have reported its sleep-promoting effect. This study aims to test the potential of HSF extract as functional food that improves sleep in humans.

**Methods and analysis:**

Eighty participants with sleep disturbances who meet the inclusion/exclusion criteria will be enrolled in this study. Since the effect of HSF extract on sleep is considered to be that of a functional food rather than a medicine, participants with severe insomnia will be excluded from the study. The enrolled participants will be randomly assigned to the HSF extract or placebo groups in a 1:1 ratio. The HSF extract and placebo capsules will look identical, and participants, investigators, and outcome assessors will be blinded to the allocation. Four capsules of HSF extract or placebo will be orally administered 30–60 min before bedtime for 4 weeks. The primary outcome of this study will be the change in the Pittsburgh Sleep Quality Index (PSQI) global score from the baseline after 4 weeks. The subjective and objective changes in the participants’ sleep will be evaluated using the Insomnia Severity Index (ISI), Epworth Sleep Scale (ESS), sleep diary, and polysomnography (PSG). The occurrence of adverse events will be closely monitored.

**Discussion:**

The results of this trial will provide data on the efficacy and safety of HSF extract in enhancing sleep quality. Based on the results, the potential of HSF extract as a functional food that improves sleep in humans will be evaluated, and the findings of the trial will be submitted to the Korean Ministry of Food and Drug Safety for consideration as a new functional ingredient that may help to improve sleep quality.

**Clinical trial registration:**

: Clinical Research Information Service: KCT0007314; Registered 19 May 2022, https://cris.nih.go.kr/cris/search/detailSearch.do/21497.

## 1. Introduction

Sleep is essential factor for maintaining physical and psychological well-being (1). Poor sleep has been found to be associated with reduced productivity and impaired work performance (2, 3), and insufficient sleep has been linked to an increased risk of cognitive decline (4). Despite the importance of sleep, dissatisfaction with sleep quantity or quality is prevalent among the general population, with up to 41.7% of individuals reporting insufficient sleep and 30–48% reporting difficulties with sleep initiation or maintenance (5, 6). Although medication may be effective for treating severe insomnia, there is still an unmet need for safe and accessible sleep aids for individuals with suboptimal sleep quality (7). One potential solution is the use of functional foods, which contain bioactive compounds that offer health-promoting properties (7, 8).

Several functional foods have been suggested as potential aids for improving sleep quality (7, 9, 10). For example, valerian, one of the botanical dietary supplements sold in the United States as sleep aids (11, 12), and chamomile, which is commonly consumed as a tea, have been reported to have beneficial effects on sleep quality (13, 14). In Korea, five functional food ingredients have been granted health claims for “may help to improve sleep quality” (15), including Ecklonia cava ethanol extract (16, 17), milk protein hydrolysate (Lactium) (18), rice bran ethanol extract (19), fermented L-glutamate GABA powder (20), and ashwagandha extract (21). The health functionality of these ingredients was established based on their sleep-promoting effects demonstrated in human and animal studies.

*Hibiscus syriacus* L. flower (HSF) is a food ingredient (22) that has been widely used to make flower tea. The root of *Hibiscus syriacus* L. is used as a medicine, and its effects on wound healing (23), depression-like behaviors, and neuroprotection (24) have been reported. Meanwhile, HSF has shown a sleep-promoting effect in three sleep-related animal models (25). The HSF extract and its active component (saponarin) increased the rapid-eye-movement sleep time in an electric foot shock-induced sleep disturbance model, restored sleep duration in a restraint-induced sleep disturbance model, and increased sleep maintenance time in a pentobarbital-induced sleep model (25). Based on the results of previous animal studies, the effect of HSF extract on sleep improvement needs be tested in humans.

The objective of this randomized, double-blind, placebo-controlled, parallel-group clinical trial is to evaluate the efficacy and safety of HSF extract on sleep improvement in adults with sleep discomfort and to compare them with that of placebo. Eighty participants will be randomly allocated to the HSF extract or placebo groups in a 1:1 ratio. The superiority of the HSF extract over placebo will be tested.

## 2. Methods and analysis

### 2.1. Study setting

This clinical trial will be conducted in two academic university hospitals in Korea, one in Seoul and the other in Daegu.

### 2.2. Eligibility criteria

This trial targets those individuals with subjective sleep complaints who are in their sub-health status, not the disease. Patients with severe insomnia will be excluded from the study.

#### 2.2.1. Inclusion criteria

Participants aged between 19 and 65, with a Pittsburgh Sleep Quality Index (PSQI) global score of 5 or higher, Insomnia Severity Index (ISI) score between 8 and 21, and who agree to participate and sign the informed consent form will be included.

#### 2.2.2. Exclusion criteria

The exclusion criteria are as follows: participants with severe diseases in cardiovascular system, immune system, respiratory system, gastrointestinal/hepatic and biliary system, kidney and urinary system, nervous system, musculoskeletal system, infectious disease, or malignant tumor; with diseases or symptoms that could affect sleep, such as nocturia; with mental diseases, including alcohol-use disorder, major depressive disorders, generalized anxiety disorders, post-traumatic stress disorders, and obsessive–compulsive disorder; with a past history of or currently suffering from schizophrenia or bipolar disorder; with cognitive decline; with sleep disorders including insomnia, narcolepsy, obstructive sleep apnea, and restless legs syndrome; with irregular sleep patterns due to night-shifts; current smokers, heavy caffeine drinkers, or excessive alcohol drinkers; those taking medications or dietary supplements that could affect sleep within 4 weeks. To exclude participants with clinical depression and anxiety, the Patient Health Questionnaire-9 (PHQ-9) (cut-off: 10 points) (26) and Generalized Anxiety Disorder-7 (GAD-7) (cut-off: 5 points) (27) will be used for the screening test. To exclude participants with a high risk of sleep apnea, STOP-Bang Sleep Apnea Questionnaire (cut-off: 5 points) (28) will be used for the screening test. The complete exclusion criteria with detailed information can be found in the clinical trial registration.^1^

### 2.3. Intervention

Four capsules containing HSF extract or placebo will be orally administered 30–60 min before bedtime for 4 weeks. The investigational products are manufactured as brown-colored hard capsules. One HSF extract capsule (500 mg) includes 250 mg of HSF extract as an active ingredient. The participants in the HSF extract group will take 1,000 mg of HSF extract per day. Placebo capsules have an appearance identical to that of the HSF extract capsules and contain maltodextrin and no active ingredients.

Discontinuing or modifying the allocated intervention is not planned, except in case of the participant’s refusal. To monitor adherence, the pharmacists will check for compliance at every visit after prescribing the HSF extract or placebo capsules, and all the remaining capsules will be returned to the clinical pharmacies. During the study period, concomitant care or interventions will be prohibited, except in exceptional cases. Only those medications, dietary supplements, and other treatments initiated before participation in this study that will not affect the results of this study can be permitted. Furthermore, even during the course of the study, if the administration of a medicine is required for the transient treatment of other diseases and the medication is considered to not affect the result, it can be permitted.

### 2.4. Outcomes

#### 2.4.1. Primary outcome

The primary outcome of this study is the change from the baseline of the PSQI (29, 30) global score after 4 weeks of administration. The PSQI is a representative self-rated questionnaire that assesses sleep quality and disturbances for over a month. A higher PSQI global score indicates poor sleep quality.

#### 2.4.2. Secondary outcome

**Figure 1:**
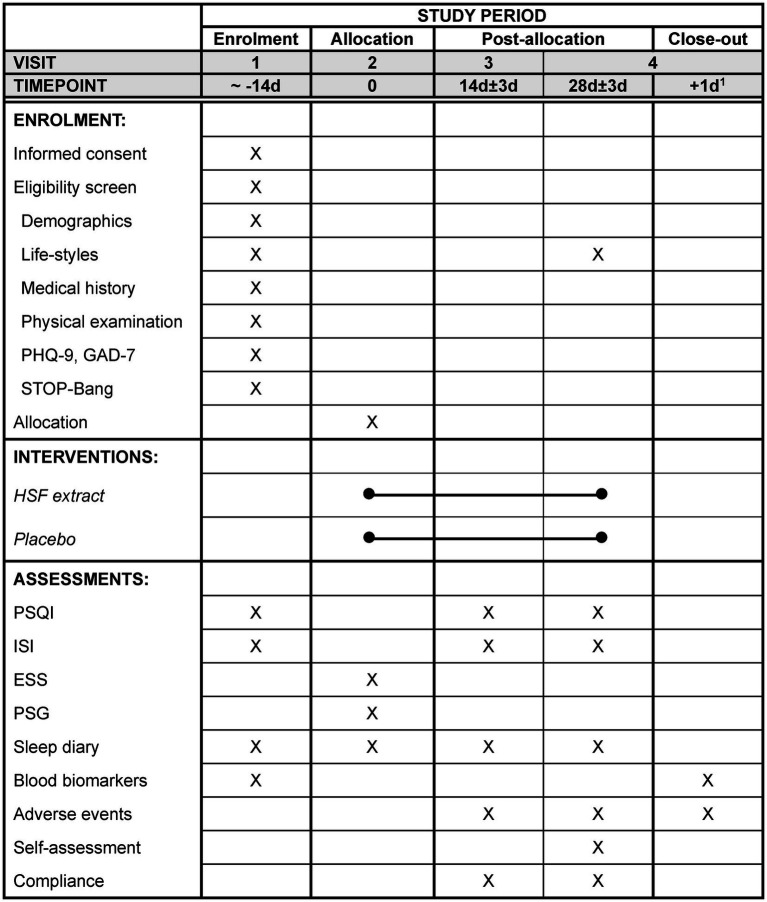
The schedule of enrolment, intervention, assessment, and visits for participants. ^1^The day after the polysomnography. PHQ-9, Patient Health Questionnaire-9; GAD-7, Generalized Anxiety Disorder-7; PSQI, Pittsburgh Sleep Quality Index; ISI, Insomnia Severity Index; ESS, Epworth Sleepiness Scale; PSG, Polysomnography.

The secondary outcomes include the ISI (31, 32) and Epworth Sleepiness Scale (ESS) (33, 34) to assess the severity of insomnia and daytime sleepiness, respectively. The ISI and ESS will be measured at baseline, 2 weeks (during treatment), and 4 weeks (post-treatment). Polysomnography (PSG) will be performed at baseline and at 4 weeks (post-treatment) for one night, and the total sleep time (TST), sleep efficiency (SE), sleep onset latency (SOL), and wake-up after sleep onset (WASO) will be assessed. The participants will be instructed to keep a sleep diary during the study period. The TST, SE, SOL, and WASO for 7 days will be extracted from the sleep diary at baseline, 2 weeks, and 4 weeks. Blood biomarkers of cortisol, C-reactive protein, and adiponectin will be measured at baseline and at 4 weeks to assess the stress levels and inflammatory markers. The participant timeline is shown in Figure 1.

### 2.5. Sample size

The primary objective of this clinical trial is to evaluate the superiority of the PSQI global score improvement in the HSF extract group compared to that in the placebo group. To estimate the mean difference of the PSQI global score between both groups and the pooled standard deviation, the results of a previous randomized trial (35) will be used to assess the PSQI global score. In the earlier trial, the mean difference of change from baseline between the two groups was −3.95 (−3.96 in the treatment group and − 0.01 in the placebo group). The standard deviation of the change from baseline was not presented, and the largest value of the standard deviation of the PSQI global score was 3.4. To calculate the sample size for this study, the mean difference and pooled standard deviation were estimated as −2.50 and 3.4, respectively. With a 5% level of significance, 80% power of the test, and 25% dropout rate, the required sample size for each group was calculated as 40.

### 2.6. Recruitment

Adults with sleep disturbance are being recruited based on the outpatients of two university hospitals in Korea. The clinical sites are located in two metropolitan cities in Korea: Seoul and Daegu. A poster regarding this clinical trial is being posted on the bulletin board and internet homepage of both the hospitals. To enhance the recruitment rate, local advertisements can also be conducted at the bus stops near the hospitals.

### 2.7. Randomization and allocation concealment

An independent statistician generated the random allocation sequence using the randomization program of the SAS^®^ system (version 9.4). The allocation ratio for the HSF extract and placebo was 1:1, and the block size was concealed from the other investigators. An investigator affiliated with Dongkook Pharmaceutical, who is in charge of the management of the random allocation sequence and packaging of investigational products, packed the HSF extract capsules and placebo capsules according to the allocated random number. The investigational products were packed identically, and each random number was labeled in the package. The investigators in charge of the enrollment of participants were blinded for the allocation sequence and sequentially assigned the random numbers to the enrolled participants.

### 2.8. Blinding

The participants of the trial, investigators, and outcome assessors will be blinded to the group assignment. Placebo and HSF extract capsules have been developed to have an identical appearance. Concealed envelopes containing information on the group assignment of each random number are being managed by the principal investigators at the two sites. These concealed envelopes will not be disclosed until the completion of this clinical trial unless unblinding is inevitable owing to serious adverse drug reactions or important clinical issues. In such cases, the investigators should immediately contact the sponsors and provide detailed information about the situation. The sponsors and investigator, only after thorough examination, will decide whether or not code breaking for the participant is necessary. The process and reason for the unblinding will be recorded, and the unblinded participants will be dropped from the trial.

### 2.9. Data collection methods

The validated Korean versions of the PSQI (30), ISI (32), and ESS (34) will be used. The PSQI is a self-rating questionnaire, and the investigators will check the answers of the participants every time to determine that they understand the question and respond properly and also to identify if a missing value or error exists. Sleep indicators extracted by PSG are also important outcomes of this trial. Qualified investigators will interpret the results of PSG. To minimize the difference among the assessors, the raw PSG data obtained at the two sites will be gathered at one site, which will take responsibility for interpreting the PSG results.

In the screening process, the PHQ-9 (26) and GAD-7 (27) will be measured to exclude participants with clinical depression or anxiety. In addition, the STOP-Bang (28) will be used to exclude participants with a high risk of obstructive sleep apnea. Validated Korean versions of the PHQ-9 (36), GAD-7 (37), and STOP-Bang (38) will be used. The investigators will send messages to participants to inform them of the dates of visits and encourage them to complete follow-ups.

### 2.10. Data management

The data obtained from the source documents, such as questionnaires, worksheets, and medical records, will be entered into an electronic Case Report Form (eCRF). Using the validation data system of eCRF, data ranges are set for each value, and a query will appear when outlier data are entered. Full Source Data Verification (SDV) for data entered into the eCRF will be conducted during routine monitoring visits. In addition, prior to trial initiation, a data management plan has already been prepared. The system query of the eCRF is planned to be checked bimonthly.

### 2.11. Statistical methods

#### 2.11.1. Definition of the population to be analyzed

The main population to be analyzed for evaluating efficacy is the Per Protocol (PP) set, and Full Analysis (FA) set will be used supplementary. The PP set will include participants who complete the trial without major deviations from the planned protocol. The FA set will include an additional population for evaluating the efficacy and will include participants who are randomized into groups and who complete more than one assessment after the administration of the investigational products. The main analysis population for evaluating safety will be the safety set, which includes participants administered the investigational products more than once. In the primary and secondary outcomes, there will be no missing data in the PP set, and missing data in the FA set will be imputed using the last observation carried forward method.

#### 2.11.2. Statistical method for analyzing primary and secondary outcomes

The change from the baseline PSQI global score at 4 weeks between the two groups will be compared using a two-sample *t*-test or Wilcoxon rank sum test, according to the normality of data. Additionally, changes in the PSQI global score over time in each group will be compared using a paired *t*-test. A generalized linear model (GLM) with covariates of caffeine consumption, age, body mass index, smartphone usage time, and drinking habits can also be conducted. In case of significant differences in baseline characteristics between the two groups, these baseline characteristics can be considered as covariates in conducting the GLM.

The statistical method used for analyzing secondary outcomes is identical to that used to analyze the primary outcome.

### 2.12. Data monitoring and auditing

The estimated risk of the trial is low because the investigational product of this trial is a food. A data monitoring committee is not required and an interim analysis is not planned.

A contract research organization hired by sponsors will monitor the clinical trial to verify the process of informed consent acquisition, conduct the trial in compliance with the approved protocol, and obtain accurate and complete trial data. The initial monitoring visits for each site are scheduled within 7 days of the enrollment of the first participant. Routine monitoring will also be conducted 10–11 times at each site.

### 2.13. Harms

Adverse events will be carefully assessed through non-directive questioning during the trial period. The symptoms and signs of adverse events in participants will be collected at every visit, and their severity and causality will be assessed. Blood and urine tests will be conducted before and after the intervention, and clinically significant abnormal results will be assessed. Owing to the characteristics of the investigational product as a health functional food, product-related severe adverse events are not expected, and mild dyspepsia may occur.

## 3. Discussion

This clinical trial aims to test the potential of HSF extract as functional food for sleep improvement in humans. Approximately one-third of the general population has low-quality sleep, whereas approximately 6–15% of the population is diagnosed with insomnia disorders (5). Some people who complain of insomnia may not require sleep medicine if their symptoms are not severe enough, and may instead benefit from functional food that can help improve their sleep quality. This protocol was developed to evaluate the functionality of HSF extract in improving sleep quality in that population. Patients with severe insomnia, depressive or anxiety disorders, and other sleep disorders are set to be excluded from this trial. Furthermore, a large effect size of health functional foods may not be obtained, and we have tried to exclude confounding factors as much as possible. Strict exclusion criteria will be applied, and participants with irregular sleep patterns due to night shifts, smoking, heavy caffeine or excessive alcohol consumption are set to be excluded. In addition, the eligibility criteria and outcomes in this trial have been planned according to the Korean MFDS functionality test guideline for health functional food that “may help to improve sleep quality” (39).

Regulations governing functional foods differ between countries. In the United States, there are three categories of health-related claims: nutrient content claims, structure/function claims, and health claims (40). However, sleep-related statements are not included in the health claims category. For instance, while valerian-containing products are promoted as sleep aids (12), they fall under the structure/function claims category and must carry a label indicating that they are not intended to diagnose, treat, cure, or prevent any disease (40, 41). In Japan, there are three types of functional foods: those with nutrient function claims, those with specified health uses, and those for special dietary uses. Since 2015, sleep-related health claims have been added to new functional products, making them one of the major health claims (42).

In Korea, functional ingredients are divided into two categories: those that have been notified by the Ministry of Food and Drug Safety (MFDS) and those that undergo individually recognized by the MFDS (43). The former includes 95 types of functional ingredients, such as vitamins, minerals, essential fatty acids, protein, dietary fiber, ginseng, and green tea extract (44). For an ingredient to obtain individual recognition as a functional ingredient, the applicant should submit data on the ingredient’s safety and functionality. The MFDS then reviews the data consults with the Health Functional Food Deliberation Committee before making a decision on recognition (45). “May help to improve sleep quality” is one of the health claims included in the individual recognition as a functional ingredient category. This trial aims to test the potential of HSF extract to obtain individual recognition for improving sleep quality.

This study protocol has some limitations. First, this is the first human study on the effect of HSF extract on sleep improvement, and no previous study has calculated the effect size of HSF extracts. Therefore, we estimated the effect size of HSF extract on sleep improvement based on the results of a previous randomized trial with a similar design. Second, there are some limitations to measuring sleep using PSG in the hospital for a single night (46, 47). Although an actigraphy device would be a good option for monitoring sleep patterns in a home environment, we could not select it due to budget constraints. To complement this, a sleep diary will be maintained and various sleep characteristics extracted from both PSG and the sleep diary will be considered. We will collect a wide range of data regarding sleep using questionnaires, PSG, and blood biomarkers; this is one of the strengths of this trial. Third, due to a delay in administrative procedures, this protocol was registered on the international clinical trials registry platform after the first participant enrolled. Nevertheless, the protocol was registered at the early stage of the trial, and the registered protocol version is the same as the version at the time of enrollment of the first participant.

## 4. Ethics and dissemination

### 4.1. Research ethics approval

This protocol was approved by the Institutional Review Boards (IRB) of Kyunghee University Hospital at Gangdong (IRB No. KHNMC 2021–11-024) and Keimyung University Dongsan Hospital (IRB No. DSMC 2021-11-07). In case protocol amendments are required, before applying the changes, approval from both the IRBs will be obtained for the revised version of the protocol. The current version of the protocol is 1.2 (date: 2022-01-10).

### 4.2. Consent or assent

Prior to the screening process, investigators delegated by the principal investigators will provide sufficient information on this clinical trial, and participants will have adequate time to decide whether or not to participate in this clinical trial. If a person decides to participate voluntarily, written informed consent will be obtained from them.

### 4.3. Confidentiality

To protect the confidentiality of participants, the initial and not the name of each participant will be entered into the eCRF, and each participant will be identified by the screening number of this trial. The two sponsoring institutes (Korea Institute of Oriental Medicine and Dongkook Pharmaceutical) will have access to the final trial dataset, and a blind review will be conducted to confirm the analysis set before the data is locked.

### 4.4. Dissemination policy

The research findings will be submitted to and published in peer-reviewed journals. In addition, the efficacy and safety results of this trial will be submitted to the Korean MFDS for individual recognition of the new functional ingredient as one that “may help to improve sleep quality.”

## Ethics statement

The studies involving human participants were reviewed and approved by the Institutional Review Board of Kyunghee University Hospital at Gangdong and the Institutional Review Board of Keimyung University Dongsan Hospital. The patients/participants provided their written informed consent to participate in this study.

## Author contributions

ML, YP, CY, DK, and KL have made contributions to the conception and design of the work. YC has drafted the manuscript. All authors substantively revised it, read, and approved the final manuscript.

## Funding

This research was supported by a grant from the Korea Institute of Planning and Evaluation for Technology in Food, Agriculture and Forestry (IPET) through the Technology Commercialization Support Program, funded by the Ministry of Agriculture, Food and Rural Affairs (MAFRA) (821020-3).

## Conflict of interest

ML was holding a patent for *Hibiscus syriacus* L. extract on improving sleep disturbance. YP, DK, and KL were employed by Dongkook Pharm. Co., Ltd.

The remaining authors declare that the research was conducted in the absence of any commercial or financial relationships that could be construed as a potential conflict of interest.

## Publisher’s note

All claims expressed in this article are solely those of the authors and do not necessarily represent those of their affiliated organizations, or those of the publisher, the editors and the reviewers. Any product that may be evaluated in this article, or claim that may be made by its manufacturer, is not guaranteed or endorsed by the publisher.

## Footnotes

^1^ https://cris.nih.go.kr/cris/search/detailSearch.do/21497
